# Safety Evaluation of Omniscan: An Observational Postmarketing Surveillance

**DOI:** 10.7759/cureus.89184

**Published:** 2025-07-31

**Authors:** Harsh Mahajan, Nafisa S Batta, Pravin Vaze, Pratibha S Shad, Kabir Mahajan, Mark G Hibberd, Burkhard Roessink, Dustin M Dunham, Jasmeet Sahni, Rohini Pandey

**Affiliations:** 1 Radiology, Mahajan Imaging Pvt Ltd, Delhi, IND; 2 Radiodiagnosis, Mahajan Imaging Pvt Ltd, Delhi, IND; 3 Medical Affairs, GE HealthCare, Dallas, USA; 4 Pharmacovigilance, GE HealthCare, Oslo, NOR; 5 Medical Affairs, GE HealthCare, Gurugram, IND

**Keywords:** adverse event, ce-mri, gadolinium-based contrast agent, postmarketing drug surveillance, safety study

## Abstract

Objective: This study aimed to conduct a single-arm, open-label, multicenter postmarketing surveillance study to evaluate the safety of Omniscan immediately and six weeks (± two weeks) after injection.

Methods: Three sites in India that use Omniscan (gadodiamide; GE Healthcare, Chicago, IL) as a contrast agent were selected. Omniscan is a linear, nonionic, nonprotein-binding gadolinium-based contrast agent. A total of 150 study participants were enrolled for contrast-enhanced magnetic resonance imaging (CE-MRI). All patients were asked for permission to be contacted six weeks (± two weeks) after CE-MRI, and participants were telephonically followed up by a healthcare professional with a questionnaire.

Results: Of the 150 enrolled participants, 133 (89%) completed the telephonic follow-up, scheduled for six weeks (± two weeks) after injection. Of these 133 participants, a total of seven (5.26%) treatment-emergent adverse events (TEAEs) were reported. Out of these seven TEAEs, one TEAE (0.007%) was serious, with seriousness criteria life-threatening. A single event was severe; the remaining adverse events (AEs) were of mild severity. None of the AEs was assessed as related to Omniscan.

Conclusions: There were no AEs causally related to Omniscan (gadodiamide) immediately after injection and during telephonic follow-up after six weeks (± two weeks). Safety analysis in this postmarketing surveillance did not reveal any increase in the safety risk of Omniscan. No AE persisted for four weeks or more after injection of Omniscan for MRI. CE-MRI with Omniscan was thus generally considered safe and well-tolerated.

## Introduction

Contrast-enhanced magnetic resonance imaging (CE-MRI) is a valuable and routine imaging technique that utilizes gadolinium-based contrast agents (GBCAs) to enhance the quality of magnetic resonance imaging (MRI) images. The rate of MR-enhanced imaging is 30% overall, and it varies significantly based on the procedure and the organ system to be visualized [1]. The intravenously injected GBCA shortens the relaxation times of hydrogen nuclei in body tissues, thereby increasing their signal intensity on T1-weighted sequences. Contrast-enhanced imaging improves image quality, investigative performance, and diagnostic confidence of the radiologist. A variety of GBCAs are available and differ across a multitude of parameters, including ionicity, osmolality, viscosity, relaxivity, molecular structure (linear or macrocyclic), and approved dosage strengths and indications. While CE-MRI is a routine mainstay of diagnostic imaging, gadolinium in its free form is a toxic rare earth metal that needs to be bound to chelating molecules to facilitate its clearance from the human body and thus minimize the risk of harmful effects. Although rare, safety risks of CE-MRI generally encompass three domains: nephrogenic systemic fibrosis (NSF), gadolinium retention in brain and other tissues, and acute adverse reactions. NSF is a progressive, multiorgan fibrosing condition mainly caused by exposure to GBCAs used in MRI [2] in patients with acute renal failure and with severe chronic kidney disease with an estimated glomerular filtration rate (eGFR) of less than 30 mL/minute/1.73 m^2^ [3,4]. Primarily characterized by thickening of the skin and subcutaneous tissue, the clinical features of NSF may involve any fibrous tissue in the body, affecting the internal organs such as the liver, heart, muscles, and lungs [5-7]. In a case series of nine patients with end-stage renal disease undergoing MR angiography, cases reported with nephrogenic fibrosing dermopathy became apparent approximately two to four weeks after administration of GBCA; the remaining four remained unaffected [8]. The recognition of this condition led to new recommendations on the use of GBCAs, especially in patients with severe chronic kidney disease, which led to changes in product information, including the Summary of Product Characteristics (SmPC) [9] with an associated warning; the occurrence of NSF has been virtually eliminated. In 2014, concerns were raised that a small amount of gadolinium from GBCAs [10] was left behind in the brain and various organ tissues for months or years, even in patients with normal renal function. Significant investigations have shown that all GBCAs can lead to gadolinium deposition in the brain, and most clinical studies have indicated that linear GBCAs had more detectable gadolinium deposition than macrocyclic GBCAs [10]. Gadolinium could deposit in the brain and other organs, but the clinical implications of gadolinium deposition in the brain remain unclear. Few symptoms, such as muscle and joint pain, brain fog, headache, and neuropathic pain, have been suggested to be associated with Gadolinium exposure, known as Symptoms Associated with Gadolinium Exposure; however, this remains unclear and is weakly evidenced [11]. Long-term retention of chemical forms of gadolinium within the tissues of patients with normal kidney function highlights the complex and incompletely understood biodistribution of GBCAs. In 2017, the United States Food and Drug Administration (USFDA) evaluated the existing data and found no scientific evidence to suggest a causal association between GBCA exposure and the development of symptoms in patients with normal renal function. The third risk with GBCAs is that of acute adverse reactions and hypersensitivity responses. While rare and usually mild, such adverse reactions can be life-threatening. An interesting paradox of clinical concerns is observed with data suggesting the lowest rate of immediate allergic adverse events (AEs) with use of the nonionic linear GBCA like Omniscan (gadodiamide) in comparison with those of ionic linear or nonionic macrocyclic GBCAs [12]. Ultimately, healthcare providers should acknowledge that no single type of contrast agent is ideal for every setting, and a multicriteria decision analysis is advantageous when considering how to best tailor GBCA selection to a patient’s specific clinical presentation and needs. Given the importance of GBCAs in routine radiological procedures and the growing global concerns related to gadolinium deposition in patients undergoing GBCA-enhanced MRI procedures, this active pharmacovigilance surveillance study was requested by the Regulatory Authority of India. This surveillance study was aimed to assess/evaluate patient safety associated with contrast-enhanced MRI exams performed with Omniscan (gadodiamide) in Indian patients in a real-world setting. Evaluation of NSF was not included in the scope of the study.

## Materials and methods

**Figure 1:**
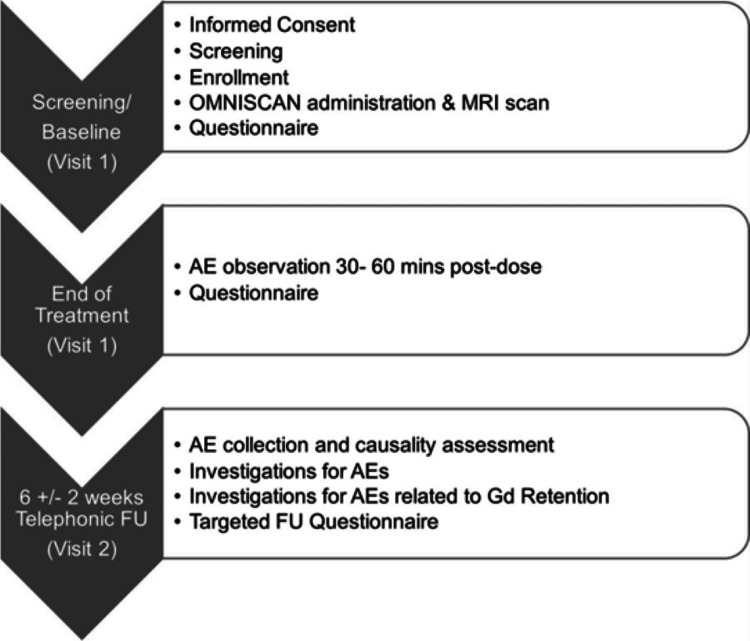
Schematic of the study FU: follow-up; MRI: magnetic resonance imaging; AE: adverse event

This was a single-arm, multicenter postmarketing surveillance study in GBCA-naïve male/female patients of at least 18 years of age, who are eligible for CE-MRI with Omniscan. Three sites in India that frequently used Omniscan (i.e., Omniscan was available for routine clinical use) and had good patient inflow were selected for this study. Patients under 18 years of age were excluded due to perceived limitations in recording follow-up responses. The protocol and informed consent form for the recruitment of subjects were reviewed and approved by an Independent Ethics Committee. A total of 150 patients (83 men and 67 women) were enrolled. The study consisted of doing CE-MRI in eligible patients with a telephonic follow-up within approximately six weeks (± two weeks) from day 1. The total enrollment duration was approximately nine months after the first patient’s enrollment at each site (Figure 1).

All available marketed treatments (prior and concomitant medications), in the investigator's judgment, were permitted during the study duration. Any other drugs/procedures that, in the investigator’s judgment, were liable to interfere or interact with the safety of the GBCA administration were excluded.

Patients were invited to participate only after the decision to use Omniscan-enhanced MRI was made. All patients consented to the active surveillance and to a follow-up six weeks (± two weeks) after Omniscan-enhanced imaging. Patients were interviewed by a healthcare professional (HCP; site personnel) according to a predecided questionnaire for collecting safety information after GBCA administration and during follow-up. HCPs at each site were trained on Omniscan safety and applicable pharmacovigilance requirements.

A complete medical history for the prior year was recorded for each patient. Vital signs, including systolic blood pressure, diastolic blood pressure, and pulse rate, were measured, and the latest available result for eGFR or any other renal function test was also recorded.

The demographic details, medical history and concurrent conditions, concomitant medications, treatments, and indication for the current MRI for all the eligible patients were collected at the baseline visit. As patients with prior exposure to GBCAs were not eligible for recruitment in the study, a confirmation was taken from the patients regarding the same during the screening/baseline visit. Vital assessments were also performed prior to the GBCA administration. All participants were administered Omniscan dose commensurate with approved Prescribing Information: 0.2 mL/kg (0.1 mmol/kg).

Out of these 150 participants, 54% participants underwent brain/spine imaging, 10% participants underwent abdomen/pelvis imaging, 12% participants underwent prostate MRI, 6% participants underwent face/neck imaging, 2% participants underwent cardiac, pituitary and knee imaging each respectively, 3.3% of participants underwent mammography, 5.3% participants underwent limbs MRI, 1.3% of participants underwent urography, and 0.6% of participants underwent imaging of kidneys, inner ear, paranasal sinuses, fistulas, and scrotum each, respectively.

The patients were observed at the site for 30-60 minutes after the administration of Omniscan regarding the occurrence of any AEs. The follow-up visit was conducted telephonically six weeks (± two weeks) after administration of Omniscan. At least two attempts at different times/day were made to contact the patient. The purpose of this telephonic visit was to collect safety data (AEs, seriousness, severity, onset date, outcome, resolution date, if applicable, safety labs, safety medications, and medical diagnosis) and to assess the causal relationship between the AE and Omniscan administration.

Details of AEs, including date of onset, outcome, seriousness, treatment, medical diagnosis/syndrome, and causality assessments, were recorded. At the same time, the patient was present at the site on day 1 and also during the telephonic follow-up visit. For participants who could not be reached for a follow-up interview, the sponsor contacted the site to inquire about the reasons for loss to follow-up and whether there were AEs.

As an observational study, the postmarketing pharmacovigilance requirements as per Indian regulations were followed. All AEs and serious adverse events were captured on the Targeted Questionnaire (see the Appendix) and the electronic case report form. Information collected included event description, seriousness, time of onset, investigations, corrective treatment, clinician’s assessment of severity, relationship to study product, and the time of resolution/stabilization of the event. All AEs that occurred during the study (including follow-up) were documented appropriately regardless of causal relationship. The safety information was also forwarded to the GE HealthCare Pharmacovigilance team as per the processes.

## Results

Safety evaluations were performed based on the collection of information regarding the patient’s medical history, past and current medications, and a review of the selection criteria; 54% (81) participants underwent brain/spine imaging, 10% (15) participants underwent abdomen/pelvis imaging, 12% (18) participants underwent prostate MRI, 6% (nine) participants underwent face/neck imaging, 2% (three) participants underwent cardiac, pituitary, and knee imaging each, respectively, 3.3% of participants underwent mammography, 5.3% participants underwent limbs MRI, 1.3% of participants underwent urography, and 0.6% of participants underwent imaging of kidneys, inner ear, paranasal sinuses, fistulas, and scrotum each, respectively.

Assessments of safety included a review of vital signs and AEs. The data from patients included in the safety population were considered for presentation of safety endpoints and were summarized descriptively (Table 1). All the participants were contacted via telephone as per schedule after 6 ± 2 weeks. However, for 17 patients, successful contact was not established; hence, no information was received. These 17 patients were thus excluded from the per-protocol (PP) participant number. Out of seven treatment-emergent adverse events (TEAEs) reported during the study period, five TEAEs resolved on the same day, one TEAE resolved within five days, and one TEAE was still ongoing at the time of follow-up, which was related to the patient’s underlying condition of cerebrovascular accident. TEAEs characterized as intermittent require documentation of the onset and duration of each episode. None of the TEAEs required urgent medical attention/admission during the study. The data was analyzed using statistical analysis.

**Table 1:** Summary of treatment-emergent adverse events: safety population

Adverse events	Total (n = 150), n (%)
Convulsion	2 (1.33%)
Loss of consciousness	1 (0.66%)
Dysarthria	1 (0.66%)
Neurological symptom	1 (0.66%)
Hematuria	1 (0.66%)
Epistaxis	1 (0.66%)

Analysis datasets

Active Surveillance (PP) Population

**Figure 2:**
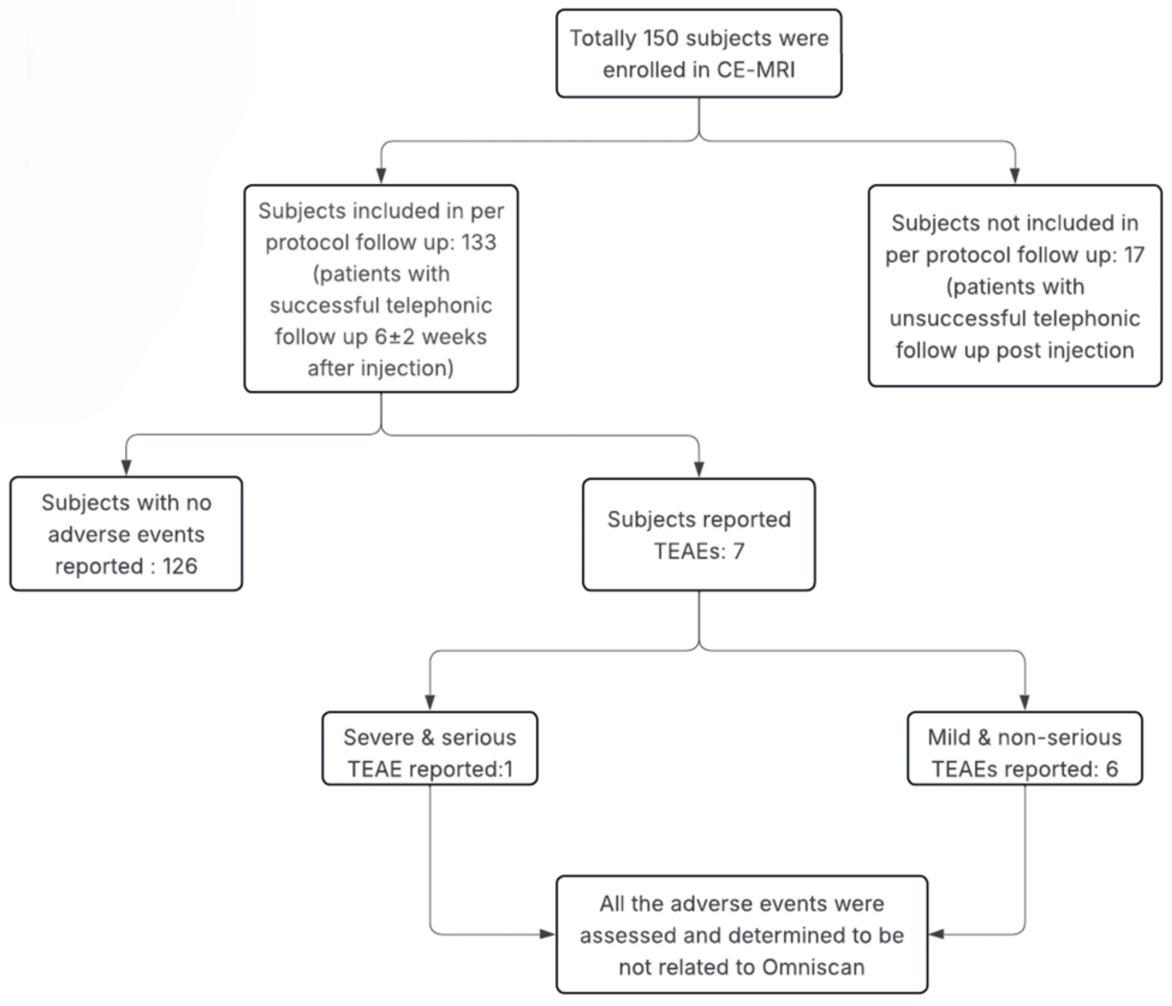
Final flowchart of the study CE-MRI: contrast-enhanced magnetic resonance imaging; TEAEs: treatment-emergent adverse events

The PP population was analyzed. The PP population consisted of all patients without any major protocol deviations, particularly those who completed the follow-up telephonic visit (Figure 2).

Safety Population

The safety population consisted of all patients who were administered Omniscan.

Statistical methods

Descriptive statistics (n, mean, standard deviation, median, and range) were provided for all the continuous variables. Frequency count (n) and percentage (%) were provided for all the categorical variables.

Out of 150 study participants, 133 (89%) completed the follow-up telephonic visit scheduled six weeks (± two weeks) after the CE-MRI with Omniscan (gadodiamide). However, all 150 participants were evaluated for any acute safety events immediately and observed for up to one hour after administration of Omniscan. None of the participants reported any acute event within 30 minutes of the administration of contrast. Out of these 133 participants who completed the active surveillance, a total of seven (5.26%) TEAEs were reported during the study. Out of the seven TEAEs, one (0.75%) was considered serious and severe. The remaining six TEAEs (4.51%) were nonserious and mild. All AEs were assessed by the investigator to be not causally related to Omniscan (Table 2). Medical history and underlying conditions of the patients provided possible explanations for the reported adverse events. No deaths were reported during the study. Out of the seven participants, five (3.75%) reported TEAEs with the system organ class (SOC) of nervous system disorders. The remaining two participants reported one (0.75%) TEAE each from the SOC of renal and urinary disorders and respiratory, thoracic, and mediastinal disorders.

**Table 2:** Subjectwise presentation of adverse events CE-MRI: contrast-enhanced magnetic resonance imaging; AE: adverse event

S no.	Gender/age	Date of CE-MRI	Adverse event start date and end date	Adverse event	Latency (interval between injection and AE onset)	Severity	Seriousness	Relatedness	Outcome
1	F/25 years	July 12, 2023	August 1, 2023, and August 1, 2023	Convulsion	20 days	Mild	No	Not related	Recovered
2	M/56 years	July 16, 2023	September 6, 2023, and September 6, 2023	Hematuria (transient)	1.5 months (July 19, 2023, to September 6, 2023)	Mild	No	Not related	Recovered
3	M/28 years	August 3, 2023	September 1, 2023, and September 1, 2023	Loss of consciousness	Approximately 4 weeks (August 3, 2023, to September 1, 2023)	Mild	No	Not related	Recovered
4	F/24 years	December 28, 2023	February 1, 2024, and unknown	Convulsion	Approximately 6 weeks (December 28, 2023, to February 1, 2024)	Mild	No	Not related	Recovered
5	M/37 years	July 26, 2023	August 1, 2023, and unknown	Dysarthria	5 days (July 26, 2023, to August 1, 2023)	Mild	No	Not related	Recovering
6	M/62 years	November 21, 2023	December 6, 2023, and December 10, 2023	Epistaxis	Approximately 15 days (November 21, 2023, to December 6, 2023)	Mild	No	Not related	Recovered
7	F/62 years	October 28, 2023	Since November 15, 2023	Increase in preexisting neurological symptoms	Approximately 20 days (October 28, 2023, to November 15, 2023)	Severe	Yes	Not related	Not recovered

## Discussion

To date, GBCAs are regarded as highly safe when administered in appropriate clinical doses as per the approved prescribing information; however, like all drugs in clinical practice, GBCAs can lead to AEs and potential risks. The incidence of allergic-like reactions to GBCAs ranges from 0.004% to 0.7%, with severe, life-threatening anaphylactic reactions ranging from 0.001% to 0.01% [13]. Most of the reactions are mild, physiological in nature, and are self-limiting, but severe anaphylactic reactions resulting in death are extremely rare and have been reported in approximately one in 300,000 administrations of GBCAs, with 40 deaths per 51 million administered GBCA doses between 2004 and 2009 [14,15]. In the present postmarketing surveillance, we did not observe any acute AE.

Although the incidence of immediate-type allergic reactions to GBCAs is thought to be low, preventing these reactions and properly managing them to minimize their adverse sequelae can improve the already exceedingly favorable GBCA safety profile [16]. Factors to consider include patients’ susceptibility to allergic reactions, skin testing, premedication, and supportive treatment of AEs. The low immediate AE rate for gadodiamide has been confirmed in several studies [17-19] and may be due to the nonionic linear structure and diminished binding/interaction with serum proteins [12]. A single-center retrospective study involving 281,945 GBCA injections (gadobutrol, gadobenate dimeglumine, gadoterate dimeglumine, and gadodiamide) found that gadodiamide (9/10,000) was associated with a lower incidence of reactions (all reactions, allergic-like reactions) compared to gadobutrol (20/10,000) and gadobenate (33/10,000) [20].

A study conducted by Kanda et al. [21] reported an association between increased signal intensity in the dentate nucleus (DN) and globus pallidus (GP) on unenhanced T1-weighted images in patients with a history of GBCA administration. Several retrospective MRI observational in vivo studies in humans demonstrated similar signal changes after multiple doses of different GBCAs [22,23]. Selected preclinical studies have also demonstrated that a large part of gadolinium was retained in the brain, bound to macromolecules in a soluble form [24,25]. These studies were followed by postmortem human and animal studies, which confirmed the presence of gadolinium in the brain after multiple administrations of GBCAs and confirmed that the MRI changes were directly related to intracranial gadolinium retention [26]. A systematic review of small retrospective studies and reports on gadolinium deposition and increased signal intensity was reported by the European Society for Magnetic Resonance in Medicine and Biology-Gadolinium Research and Education Committee. The increased signal intensity in DN and GP was associated with linear GBCA. Macrocyclic GBCA did not show an increase in signal intensity even with larger dosage [27]. It is to be noted that intact GBCA molecules do not cross the blood-brain barrier. It is considered that GBCA can reach the cerebrospinal fluid through the choroid plexus and the ciliary body. GBCA can reach the brain interstitium through glymphatic channels via the perineural sheath and perivascular spaces of cortical arteries [28].

In 2017, the USFDA evaluated the existing data and found no scientific evidence to suggest a causal association between GBCA exposure and the development of symptoms in patients with normal renal function [9]. The selective Gd retention in brain nuclei and certain other areas is still an unanswered question. It can be attributed to multiple aspects such as anatomical variations, susceptibility, and molecular configuration at certain brain structures. The glymphatic channels can act as both a mode for Gd dechelation, and if the GBCA structure is undisrupted, it can also aid in the clearance of the molecule [29,30].

Given the importance of GBCAs in routine radiological procedures, the Regulatory Authority of India sought to assess and evaluate real-world postmarketing information on the safety of gadodiamide in contrast-enhanced MRI in Indian patients, thereby necessitating the conduct of this postmarketing, active pharmacovigilance surveillance study.

In this single-arm, multicentric, open-label, postmarketing observational study, which evaluated all prospective AEs immediately post-Omniscan dose in 150 participants and six weeks (± two weeks) after Omniscan administration in at least 133 participants, a total of seven AEs were reported. All AEs were assessed by the investigator to be not causally related to the use of Omniscan as the contrast agent. It also corroborates the finding that gadolinium exposure is nonpathological in nature, and the clinical impact is still nonconclusive.

While this study offers valuable insights, certain limitations must be acknowledged. The small sample size and observational design may introduce biases and limit the broader applicability of the findings. Due to the restricted timeline of follow-up (six weeks), the study does not capture the incidence of NSF or the long-term effects of gadolinium deposition. Ongoing or published studies may provide additional insights into the safety of GBCAs.

However, the analysis of safety in this study did not determine any increase in known safety risk (ref-SmPC) of Omniscan. There were no adverse reactions (i.e., causally related AEs) that were present immediately or during telephonic follow-up after six weeks (± two weeks) or that persisted for four weeks or more after injection of Omniscan (gadodiamide).

## Conclusions

CE-MRI with gadodiamide was generally safe and well tolerated. Safety analysis in this postmarketing surveillance did not reveal any increase in safety concerns or pharmacovigilance profile associated with gadodiamide. In particular, there were no AEs causally related to Omniscan (gadodiamide) immediately after injection and at the follow-up visit after six weeks (± two weeks). No AE persisted for four weeks or more after injecting Omniscan. More prospective studies, with a larger sample size and long-term follow-up, should be planned to better understand the potential risks (if any) of Gadolinium deposition.

## Appendices

Patient questionnaire: post-GBCA-enhanced MRI follow-up

This Targeted Questionnaire is intended to capture data from a follow-up call of patients enrolled in the active surveillance. According to the protocol, a follow-up call shall take place approximately six weeks (± two weeks) after the GBCA-enhanced MRI examination.

Unless the Targeted Questionnaire itself is extended, other data from the Active PV Surveillance will be captured in a CRF or similar document.

A. Symptoms After the GBCA-Enhanced MRI

Q1. Were any adverse reactions or symptoms observed or reported after the GBCA-enhanced MRI?

☐ No

☐ Yes (If Yes, please complete the questions below)

Symptom Details (add rows as appropriate):

Symptom Description

Details

Onset Date

Resolution Date

☐ Not resolved

☐ Recovering/Resolving

☐ Recovered with sequelae (specify)

☐ Fatal

Seriousness of the symptom:

☐ Nonserious event

☐ Life-threatening

☐ Hospitalization/prolongation

☐ Medically important event

☐ Congenital malformation/birth defect

☐ Disability

☐ Death

Tests performed to evaluate the symptom (specify)

Causal relationship with GBCA:

☐ GBCA is Reasonable cause

☐ GBCA is not reasonable cause

If not GBCA, most likely cause (specify)

Treatment to correct the symptom (specify)

Medical diagnosis/syndrome (specify)

How was the diagnosis made (specify)

Tests performed to determine cause(s) (specify)

Any treatment(s) for the condition/syndrome (specify)

Overall outcome (specify)

Duration before complete recovery (specify, provide date(s)

Q2. Was the ADR causally related to Omniscan administration?

(*Investigator discretion)

☐ GBCA is reasonable cause

☐ GBCA is not reasonable cause

If not GBCA, most likely cause (specify): __________________________

B. GBCA Administration History

Q3. Was any other administration of GBCA done before this study?

☐ No

☐ Yes (If Yes, please complete the table below)

Date

GBCA Brand Name

Lot Number

Facility

Dose & Route

Procedure & Indication

MRI Results

C. Investigational Results

Elevated levels of gadolinium in the body?

(Increased signal intensity on radiological scans. If yes (and available), please specify the date and location this increased signal intensity was seen, and how many scans/total cumulative dose of GBCAs)

☐ No

☐ Yes (specify below)

Date

Lab Test Name

Facility

Location & Specimen

Gd Levels (units)

Reference Range

Increased signal intensity on radiological scans?

☐ No

☐ Yes (specify below)

Date

Scan Type

Facility

Location & Image Details

*Depending on symptoms reported, query for more specific lab tests, skin biopsy results, urine testing, etc.

D. Renal Function

Normal renal function prior to GBCAs?

☐ Yes

☐ No (specify below)

Test

Date

Result (units)

Reference Range

Estimated GFR

Creatinine Clearance

E. Medical History

Prior or concurrent conditions?

☐ No

☐ Yes (specify below):

☐ Inflammatory

☐ Immune

☐ Autoimmune

☐ Cutaneous

☐ Neurologic/psychiatric

☐ Allergies/asthma/drug reactions

☐ History of contrast agent reactions

F. Treatments

Concomitant or recent treatments (e.g., chemotherapy, radiation, parenteral therapy, etc.)?

☐ No

☐ Yes (specify indication): __________________________

G. Medications and Supplements

List all medications, multivitamins, and supplements:

Name

Dose

Start Date

Stop Date

Indication

H. Reporter Information

☐ Interviewer:

☐ Principal Investigator

☐ Radiologist

☐ Other Physician

☐ Radiology Tech

☐ Consumer

☐ Other (specify): ____________

Name: __________________________

Occupation/Title: __________________________
